# Protective Effect of Topiramate against Diabetic Retinopathy and Computational Approach Recognizing the Role of NLRP3/IL-1β/TNF-α Signaling

**DOI:** 10.3390/biomedicines11123202

**Published:** 2023-12-01

**Authors:** Hala M. F. Mohammad, Mohamed Ahmed Eladl, Asmaa K. K. Abdelmaogood, Rabie E. Elshaer, Walaa Ghanam, Abdelhakeem Elaskary, Mohamed A. K. Saleh, Amira H. Eltrawy, Sahar K. Ali, Suzan M. M. Moursi, Shymaa E. Bilasy, Sawsan A. Zaitone, Wafa Ali Alzlaiq, Hayam Atteya

**Affiliations:** 1 Department of Clinical Pharmacology, Faculty of Medicine, Suez Canal University, Ismailia 41522, Egypt; 2 Center of Excellence in Molecular and Cellular Medicine (CEMCM), Faculty of Medicine, Suez Canal University, Ismailia 41522, Egypt; 3 Department of Basic Medical Sciences, College of Medicine, University of Sharjah, Sharjah 27272, United Arab Emirates; 4 Department of Clinical and Chemical Pathology, Faculty of Medicine, Suez Canal University, Ismailia 41522, Egypt; 5 Pathology Department, Faculty of Medicine (Boys), Al-Azhar University, Cairo 11884, Egypt; 6 Department of Pathology, Faculty of Medicine, Suez University, Suez 43533, Egypt; 7 Ophthalmology Department, Al-Azher Asyut Faculty of Medicine for Men, Asyut 71524, Egyptmohamedsalih858.el@azhar.edu.eg (M.A.K.S.); 8 Department of Anatomy and Embryology, Faculty of Medicine, Alexandria University, Alexandria 21526, Egypt; 9 Department of Anatomy, Faculty of Medicine, University of Tabuk, Tabuk 71451, Saudi Arabia; 10 Department of Clinical Pharmacology, Faculty of Medicine, Zagazig University, Zagazig 44519, Egypt; 11 Medical Physiology Department, Faculty of Medicine, Zagazig University, Zagazig 44519, Egypt; 12 College of Dental Medicine, California Northstate University, 9700 Taron Dr., Elk Grove, CA 95757, USA; 13 Department of Biochemistry, Faculty of Pharmacy, Suez Canal University, Ismailia 41522, Egypt; 14 Department of Pharmacology and Toxicology, Faculty of Pharmacy, University of Tabuk, Tabuk 71451, Saudi Arabia; 15 Department of Pharmacology and Toxicology, Faculty of Pharmacy, Suez Canal University, Ismailia 41522, Egypt; 16 Department of Clinical Pharmacy, College of Pharmacy, Imam Abdulrahman Bin Faisal University, Dammam 31441, Saudi Arabia; 17 Department of Pharmacy Practice and Clinical Pharmacy, Faculty of Pharmacy, Future University in Egypt, Cairo 11835, Egypt; 18 Department of Medical Pharmacology, Faculty of Medicine, Cairo University, Giza 12613, Egypt

**Keywords:** computational analysis, diabetic retinopathy, mouse, NLRP3/IL-1β signaling, topiramate

## Abstract

The possible impact of topiramate against diabetic retinopathy (DREN) and its molecular mechanisms in relation to the nod-like receptor family pyrin domain containing 3 (NLRP3) inflammasome has not been studied before. Thus, in the present study, we aimed to utilize a computational approach to investigate the possible protective effect of topiramate on experimental DREN and explore its impact on NLRP3/interlukin-1β signaling and brain-derived neurotrophic factor (BDNF) expression. Male albino mice were distributed to four experimental groups and assigned the following categorizations: (i) saline, (ii) diabetic, (iii) diabetic + topiramate 10 mg/kg and (iv) diabetic + topiramate 30 mg/kg. We observed shrinkage of total retinal thickness and elevation in retinal glutamate, malondialdehyde, NLRP3 and interlukin-1β but decreased glutathione (GSH) levels in the diabetic mice. Additionally, retinal ultra-structures in the diabetic group showed abnormalities and vacuolations in the pigmented epithelium, the photoreceptor segment, the outer nuclear layer, the inner nuclear layer and the ganglion cell layer (GCL). Mice treated with topiramate 10 or 30 mg/kg showed downregulation in retinal malondialdehyde, NLRP3 and interlukin-1β levels; improvements in the retinal pathologies; enhanced immunostaining for BDNF and improved ultra-structures in different retinal layers. Overall, the current results suggest topiramate as a neuroprotective agent for DREN, and future studies are warranted to further elucidate the mechanism of its protective action.

## 1. Introduction

Diabetic retinopathy (DREN) is among the devastating microvascular complications of diabetes in adults of working age [1]. Oxidative stress (OXS) and inflammation have been implicated in the retinal capillary basement membrane thickening and increased retinal capillary permeability, ultimately leading to the development of DREN [2,3]. DREN is considered a microvascular disease, but neurodegeneration of the retina is also implicated in the pathogenesis. Mitigating OXS was previously reported to decrease hyperglycemia-induced neuronal damage [4]. In addition, proper glutamate metabolism was found to be crucial for the maintenance of retinal health [5]. Diabetes could contribute to glutamate accumulation in the retina, which eventually leads to the hyperexcitation of N-methyl-D-aspartate receptors (NMDARs), enhancement of calcium influx and retinal neurodegeneration [6].

The nod-like receptor family pyrin domain containing 3 (NLRP3) inflammasome is an essential mediator of host immune responses through the activation of caspase-1 and interleukin 1β (IL-1β)/IL-18 [7], which aggravates glucose intolerance and insulin resistance [8]. Murakami et al. previously reported a critical role for calcium mobilization in activating the NLRP3 inflammasome and demonstrated that blockage of calcium mobilization hinders the NLRP3 inflammasome assembly [9]. In addition, NLRP3 inflammasome activation resulted in oxidative stress and calcium signaling [10]. The elevation of cytosolic calcium was crucial for NLRP3 inflammasome activation, and inhibition of calcium signaling diminished caspase-1 stimulation and IL-1β release in response to NLRP3 signaling [11]. Sodium influx and chloride efflux were also involved in NLRP3 inflammasome stimulation. Hence, blocking the sodium influx inhibits the activation of NLRP3 inflammasome [12].

The current strategies for the management of DREN include a combination of medical interventions, laser therapy and surgical options. Systemic management of hyperglycemia, dyslipidemia and hypertension is the most vital and efficient strategy for prevention of both the development and progression of DREN [13]. At present, three drugs have been approved by the US Food and Drug Administration for DREN, namely ranibizumab, pegaptanib and aflibercept. They decrease the rate of retinal deterioration [5]. Ranibizumab and aflibercept are administered as an intravitreal injection into the eye. The injection is usually performed in a clinical setting by an ophthalmologist. The frequency and duration of treatment depend on the severity of the condition and individual patient response [14,15]. Another effective drug is pegaptanib, which is primarily indicated for the treatment of neovascular age-related macular degeneration and is not specifically approved for DREN. Other anti-vascular endothelial growth factor (anti-VEGF) agents like ranibizumab and aflibercept are preferred [16].

Topiramate, a sulfamate-substituted derivative of d-fructose, is currently used as an antiepileptic drug and approved in several countries for prophylaxis of migraine in different age groups [17,18] and was claimed to improve insulin resistance [19]. Topiramate negatively modulates the voltage-activated sodium channels, blocks AMPA and kainate glutamate receptors and negatively modulates the effects on L-type high voltage-activated calcium channels. This contributes to its anticonvulsant, antinociceptive and neuroprotective activities. Moreover, topiramate inhibits programmed cell death, which is thought to contribute to the pathogenesis of neurological diseases [20,21].

The aim of our study is to delineate a possible protective effect of topiramate against neuronal degeneration in DREN by computational approach and by an experimental mouse model and explore whether this effect may be related to inhibition of NLRP3/IL-1β/TNF-α inflammatory signaling pathway.

## 2. Materials and Methods

### 2.1. Computational Study for Potential Target Prediction of Topiramate Role in Treatment of DREN

#### 2.1.1. Topiramate Predicted Targets

Isomeric SMILES and chemical formulation of topiramate were retrieved from PubChem (https://pubchem.ncbi.nlm.nih.gov/, accessed on 7 March 2023). Genes targeted by topiramate were anticipated using STITCH (http://stitch.embl.de/, accessed on 7 March 2023), through which we screened the target genes by setting “minimal required interaction score” as ‘high confidence (0.700)’. To expand the list, another set of predicted targets was retrieved from Swiss Institute of Bioinformatics using the SwissTargetPrediction tool (http://www.swisstargetprediction.ch/, accessed on 7 March 2023). Both lists were combined, and duplicates were removed.

#### 2.1.2. Targets (Genes/Proteins) Involved in Diabetic Retinopathy

Genes/Proteins targeted in diabetic retinopathy were identified from open-access public databases. The datasets included in the analysis were obtained from Online Mendelian Inheritance in Man (OMIM, https://omim.org/, accessed on 9 March 2023), STRING database (https://string-db.org, accessed on 9 March 2023) using ‘Pathway/Process/Disease’ tool with high confidence 0.700 interaction score, DisGeNET (http://www.disgenet.org/, accessed on 10 March 2023) and Therapeutic Target Database (TTD, https://db.idrblab.net/ttd/, accessed on 10 March 2023). These revealed a total of 685 unique targets.

#### 2.1.3. Data Analysis and Visualization

FunRich software (www.funrich.org) version 3.1.3 was accessed on 12 March 2023 and used to construct a Venn diagram for visualizing the overlapping genes, while Cytoscape version 3.9.1 was used to visualize the interaction between those overlapped genes after using the ClusterViz app, which generated the clusters via MCODE and visually categorized them according to their combined score. 

#### 2.1.4. Enrichment Analysis

Two enrichment analyses were carried out. The first was to locate the cellular targets of topiramate. The second one was carried out using the list of overlapping genes between topiramate targets and diabetic retinopathy. Both were carried out using ShinyGO 0.77 software with homosapians as species and FDR cut-off set at 0.05.

### 2.2. Chemicals and Drugs

Alloxan used for induction of diabetes was purchased from SD Fine Chem Ltd., Mumbai, India. Topiramate was obtained from Tabuk Pharmaceutical Company, Riyadh, Saudi Arabia.

### 2.3. Biological Study in Mice

All experiments were performed following the ethical guidelines of the Research Ethics Committee at the Faculty of Medicine (research 5229#) at Suez Canal University (SCU). In addition, we obtained approval from the Research Ethics Committee of the Faculty of Pharmacy at Suez Canal University (202302RA8). We used males of Swiss albino mice (body weight around 22 g, *n* = 5 per group) who were randomly allocated into 4 groups. They were housed in a polyethylene cage in normal light/dark cycle with continuous free access to food and water. Strict care and hygiene measures maintained a healthy environment for the mice.

### 2.4. Induction of DM

We allocated the mice into groups:

- Group I (Saline control): Mice received intraperitoneal injections of saline (8 mL/kg).
- Group II (Diabetic control group): Mice received intraperitoneal injections of alloxan (180 mg/kg). Development of diabetes was monitored by One Touch glucometer, and it was defined by attaining blood glucose levels higher than 200 mg/dL one-week following alloxan administration [22,23].
- Groups III and IV (Diabetic mice treated with topiramate 10 and 30 mg/kg, respectively) [24,25,26].

Animals with confirmed diabetes (fasting blood glucose > 11 mmol/L were considered diabetic mice [27]. Following the development of diabetes, the mice were left for 9 weeks to allow the development of DREN. After that, topiramate administration was initiated in groups III and IV; topiramate was given by oral gavage every other day for 28 days. After finishing the drug treatments, mice were sacrificed by cervical dislocation under ketamine anesthesia. Then, the left eye of each mouse was dissected, and retinas were isolated and frozen. One part of the frozen retinas was dipped in phosphate-buffered saline (pH 7.4), homogenized using a GlasCol homogenizer and centrifuged to obtain the clear supernatants, which were then used for ELISA measurements.

### 2.5. ELISA Assay of Glutamate, NLRP3, IL-1β and TNF-α in Retinal Homogenates

Supernatants of the retinal homogenates were used for quantitative measurements of glutamate using an ELISA kit (#ab83389), IL-1β (#ab100768) and TNF-α (ab46070) obtained from Abcam (Waltham, MA, USA) based on the instructions of the manufacturer. The plates were read at 405 nm using a Stat Fax ELISA reader (model 2100, NE LabSystem, Chestnut Hill, MA, USA).

### 2.6. Assessment of Lipid Peroxidation and Reduced Glutathione (GSH)

Lipid peroxidation is frequently utilized as an indication of cell membrane damage caused by reactive oxygen species. One of the main peroxidation byproducts of polyunsaturated fatty acids is malondialdehyde (MDA), which is widely used to measure OXS in tissue samples [28]. We used Biodiagnostics spectrophotometric kit (Cairo, Egypt) based on the thiobarbituric acid assay to determine MDA concentration in the retinal homogenates [29]. Thiobarbituric acid reacts with MDA at 95 °C, producing a product that can be measured using a UV spectrophotometer at 534 nm. Second, GSH concentration was determined using the Biodiagnostics GSH assay kit (Cairo, Egypt), and the absorbance of the sample was measured at 405 nm against a tube containing a blank solution [30]. The color of the product is in direct proportion to the GSH level.

### 2.7. Western Blotting for NLRP3 and IL-1β in Retinal Homogenates

We transferred the supernatants of retinal homogenate to new microcentrifuge tubes, and 5 µL was utilized for determining the concentration of protein using assay kit from Bio-Rad (Quick StartTM Bradford Protein). We loaded analogous concentrations of proteins on a sodium dodecyl sulfate–polyacrylamide gel next to treatment with Bio-Rad 4× Laemmli Sample Buffer (Hercules, CA, USA). After separation of proteins, the gel was then poured into a nitrocellulose membrane and blocked with 5% Bio-Rad dried milk (USA) for 1 h. 

Then, we washed the blocked membranes and incubated them with gentle agitation with the selected primary antibodies against NLRP3 (1:500) and IL-1β (1:200) at 4 °C overnight. Then, we washed the blots again and incubated them with horseradish peroxidase (HRP)-conjugated secondary antibody (goat anti-rabbit IgG H&L (HRP, ab6721) and goat anti-mouse IgG H&L (HRP, ab205719). We visualized the protein bands with the enhanced chemiluminescence ECL Advance TM Western blotting detection kit (Amersham BioSciences, Buckinghamshire, UK). We quantified the intensity of immunoreactivity by densitometry using ImageJ software version 1.53m (NIH, Bethesda, MD, USA).

### 2.8. Histological Staining by Hematoxylin–Eosin and Image Analysis

The right eyes were dissected and fixed in paraformaldehyde solution for 24 h. Next, we removed the anterior segment of each eye, cleared the vitreous and left the eyecup (optic nerve kept attached to the retina) for further fixation overnight. We embedded the eyecups in paraffin blocks and started cutting multiple level serial sections from each block through the eye globe. We prepared 4–5 µm sections, deparaffinized the sections, performed a hydration step and stained the sections with hematoxylin–eosin (H-E) to identify the thickness of the retinal layers and pathological changes. Deparaffinizing retinal slides were prepared by immersing them in absolute xylene for four minutes. After being mordanted for 10 min with phosphomolybdic acid solution, they were quickly immersed in methyl blue. Then, 1% acetic acid solution was added to slides for a couple of minutes after cleaning in PBS for one minute [31]. Slides were examined under an optical microscope (OLYMPUS BX50, Tokyo, Japan). Retinal examination was performed on sections showing the whole retina at the level of optic disc and attachment of optic nerve to eye globe in order to include same level of retina on each sample. Then, the images were captured at equal distribution and investigated by using the ImageJ software (NIH, Bethesda, MD, USA). The total thickness of retinas, as well as the thickness of the ONL and INL, was calculated as the average lengths of at least six images from each animal.

### 2.9. Immunohistochemistry for Retinal BDNF

Retinal tissue sections of 4 μm were dewaxed and dehydrated through graded alcohols and covered by citrate buffer of pH = 6.0 at 92 °C for 15 min. Sections were then immersed in 3% H_2_O_2_ solution for 12 min to inhibit endogenous peroxidases. Next, sections were incubated with brain-derived neurotrophic factor (BDNF) antibody (1:100, YPA1962, Biospes Co., Ltd., Chongqing, China) overnight in the refrigerator and then washed and left to react with the secondary antibodies. 3,3′ diaminobenzidine (DAB) was utilized as a chromogen (Genemed, Torrance, CA, USA), and finally, Mayer’s hematoxylin was employed for counterstaining the tissues. We examined the slides under a light microscope, captured microscopic images and estimated the percentage of the stained area using ImageJ software version 1.54f (NIH, Bethesda, MD, USA).

### 2.10. Electron Microscopy

We cut parts of fresh retina specimen o into small pieces (0.5–1 mm^3^) and immediately fixed them in 3% phosphate-buffered glutaraldehyde (pH 7.4) at 4 °C. After that, we fixed the specimens in a freshly prepared 1% osmium tetroxide solution prepared in phosphate buffer. Next, we performed a dehydration step using ascending ethanol alcohol concentration followed by dehydration in absolute ethanol for 15 min twice. To complete the dehydration, propylene oxide was cleared and the specimens were embedded in labeled plastic capsules. Capsules were polymerized for 48 h at 60 °C. We trimmed the polymerized block into a pyramid with a small trapezoid surface and prepared semithin sections (2 μm) by LKB ultramicrotome. Then, we stained the sections with toluidine blue and inspected them with a light microscope to overview the specimens and select the best sections for further ultra-sectioning. Ribbons of ultra-thin silvery sections (0.5–1 μm) were collected. The next step was mounting the sections on copper grids and keeping them in petri dishes, followed by staining with urinal acetate for 20 min and lead citrate for 10 min. The grids were examined and photographed by TEM (Jeol 100 CX, Tokyo, Japan) at the Electron Microscopy Unit [32,33].

### 2.11. Statistical Analysis of the Data Sets

Results were arranged in tables and presented as mean ± S.D. Normality of distribution for each individual data set was compared by Shapiro–Wilk test. We compared the data that showed Gaussian distribution by one-way analysis of variance (ANOVA) and then Bonferroni’s post-hoc test. We used the GraphPad Prism program for applying the statistical tests. The *p* values were two-sided, and *p* < 0.05 was set as an acceptable level of significance.

## 3. Results

### 3.1. Topiramate Predicted Targets

The combined analysis revealed 118 unique targets after duplicate removal. Those represented the input for topiramate in the drug–disease analysis. 

### 3.2. Targets (Genes/Proteins) Involved in DREN

**Figure 1:**
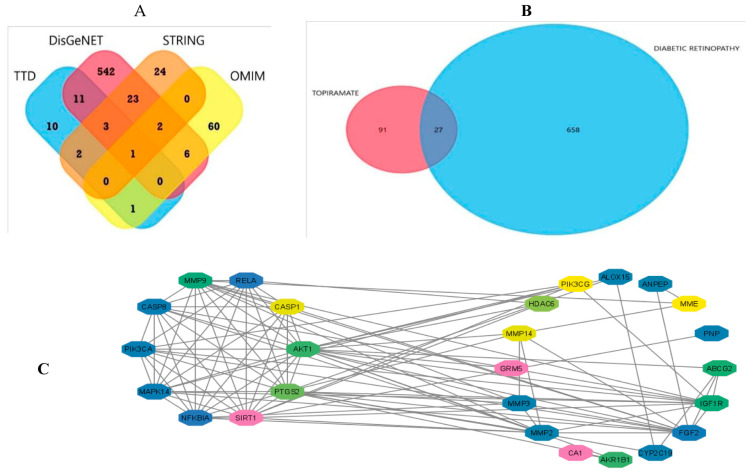
Diagrammatic presentation of the bioinformatic analysis of the unique targets in the drug–disease proposed relationship. (**A**) Venn diagram showing the overlapping genes between the four used databases (TTD; Therapeutic Drug Targets, OMIM; Online Mendelian Inheritance in Man, DisGeNet; Disease gene Network) in diabetic retinopathy. Analysis and figure construction were performed using FunRich software version 3.1.3. (**B**) Venn Diagram showing the overlapping targets of topiramate (small pink circle) and diabetic retinopathy (bigger blue circle) with duplicates removed. It revealed 27 overlapping genes from the 685 diabetic retinopathy-related genes. Analysis and figure construction were performed using FunRich software version 3.1.3. (**C**) Network with 26 nodes and 110 edges showing high interaction between the overlapped genes, including CASP1 & 8 and NF-κB. Image was generated using Cytoscape version 3.9.1.

The targets were retrieved from all data sets using the same search input (i.e., DREN). This resulted in 685 unique targets after duplicate removal, as shown in Figure 1A.

### 3.3. Data Analysis and Visualization

A Venn diagram was generated via FunRich and resulted in 27 overlapped genes between topiramate and DREN, as shown in Figure 1B. Cytoscape generated a clustered network in which the above 27 genes were used as input, and they all turned out to be highly interactive (101 edges within the network) except for one (26 genes in total, Figure 1C).

### 3.4. Enrichment Analysis

**Figure 2:**
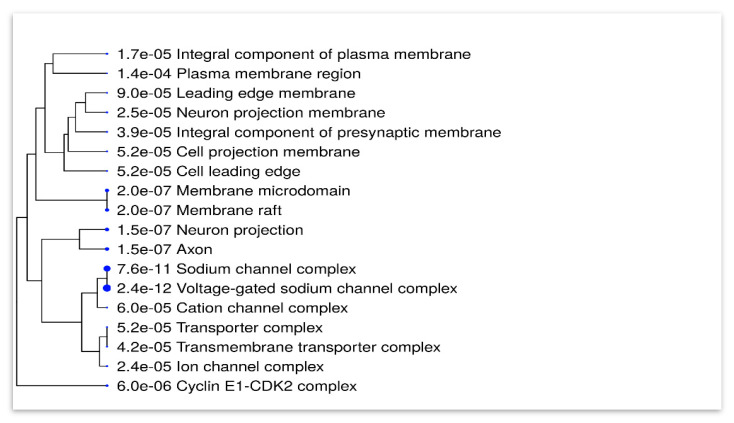
A hierarchical clustering tree that summarizes the correlation among significant pathways. Pathways of analyzed genes are clustered together according to cellular GO components with bigger dots indicating more significant *p*-values. The figure was constructed using ShinyGO 0.77 software (bioinformatics.sdstate.edu/go).

A hierarchical clustering tree was generated to summarize the cellular components of topiramate targets generated from the combined predicted analysis performed earlier, which comprised 118 targets. Surprisingly, it was highly localized in neuronal cells, ion channels and complexes. Figure 2 summarizes the correlation among significant pathways. Pathways of analyzed genes are clustered together according to cellular GO components with bigger dots indicating more significant *p*-values.

**Figure 3:**
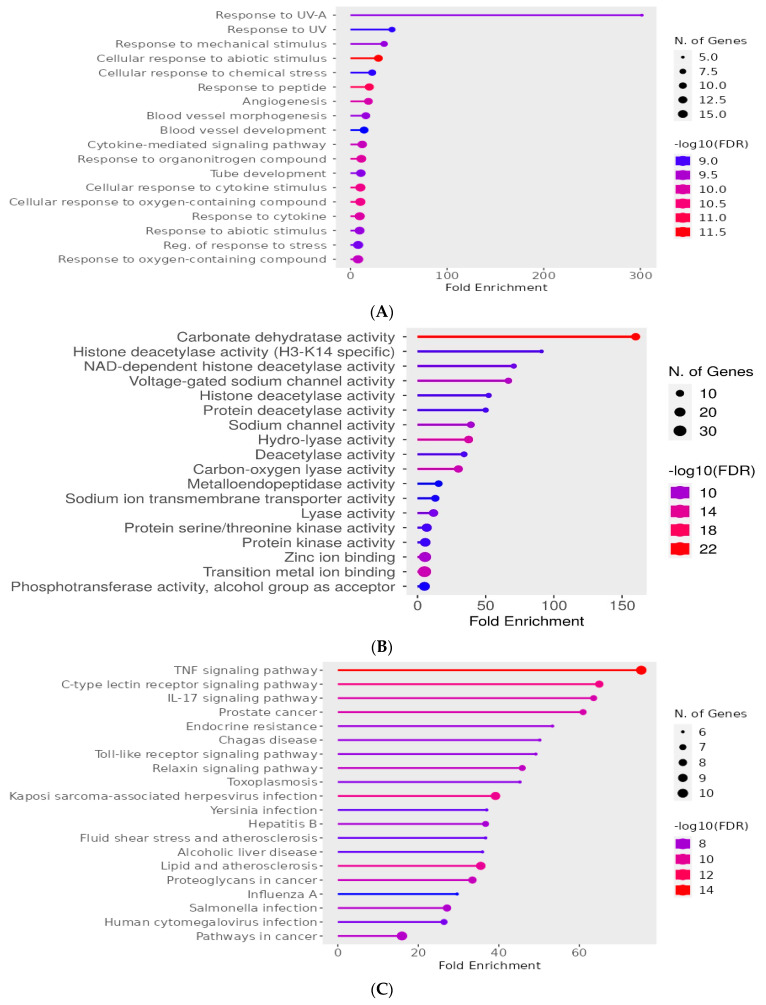
Enrichment analysis of miscellaneous signaling pathways represented as Bubble charts with bigger dots indicating more significant *p*-values. Charts were constructed using ShinyGO 0.77 software (bioinformatics.sdstate.edu/go). (**A**) Bubble Chart of 18 signaling biological pathways related to existence and development of DREN. (**B**) Bubble Chart of the top 18 GO molecular signaling pathways related to the molecular pathology of DREN and topiramate action. (**C**) Bubble Chart of the top 20 pathways from KEGG enrichment analysis of the overlapped genes to explore the pathways enriched in both the drug and the disease.

The second enrichment revealed that most of the overlapped genes are involved in biological processes related to angiogenesis, blood vessel development and response to cellular stress, all of which are hallmarks of diabetic retinopathy and are also targeted by topiramate (Figure 3A). Also, to add more evidence, the molecular GO components were analyzed and showed targets that are known to be involved in topiramate action and affected in DREN as well, such as carbonate dehydratase and voltage-gated Na^+^ channels (Figure 3B). KEGG enrichment analysis of the overlapped genes was finally performed to explore the pathways enriched in both the drug and the disease. Topiramate was involved in the IL-17 pathway, endocrine resistance, TNF signaling and lipid and atherosclerosis (Figure 3C).

### 3.5. Topiramate Protected against DREN in Mouse Study

#### Topiramate Reduced the Level of Retinal Inflammatory Mediators

**Figure 4:**
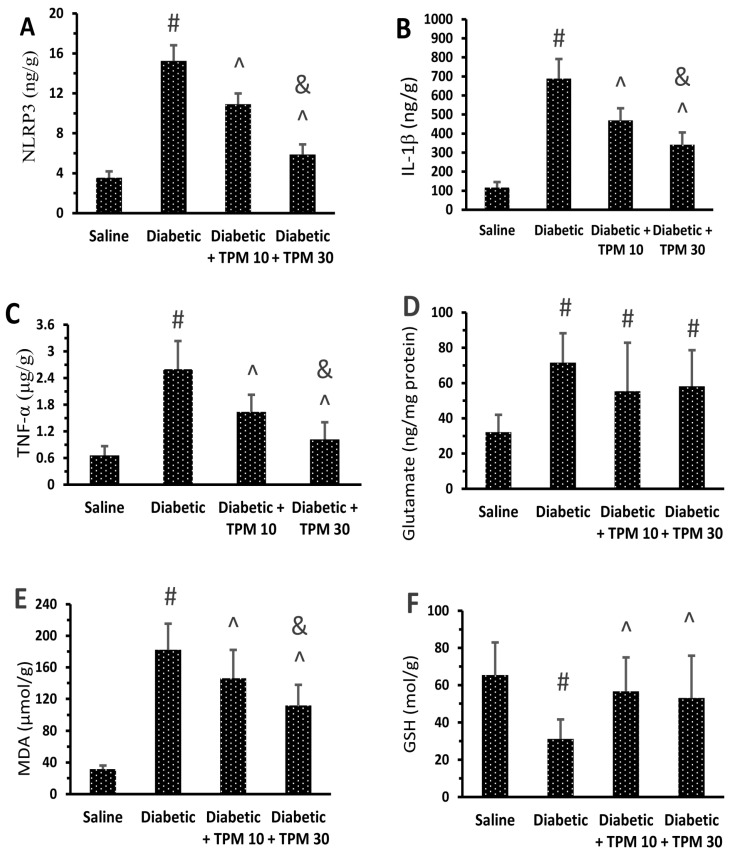
Effect of topiramate on retinal NLRP3, IL-1β, TNF-α, glutamate, MDA and GSH in the retinal homogenates (**A**–**F**). NLRP3: Nod-like receptor family pyrin domain containing 3, IL-1β: interlukin-1β, TNF-α: tumor necrosis factor-α, MDA: malondialdehyde, GSH: reduced glutathione, TPM: topiramate. Data in the figures are the means ± SD and analyzed by one-way ANOVA test and post-hoc analysis at *p* < 0.05. #, ^, &: versus saline, diabetic mice and diabetic + TPM group.

**Figure 5:**
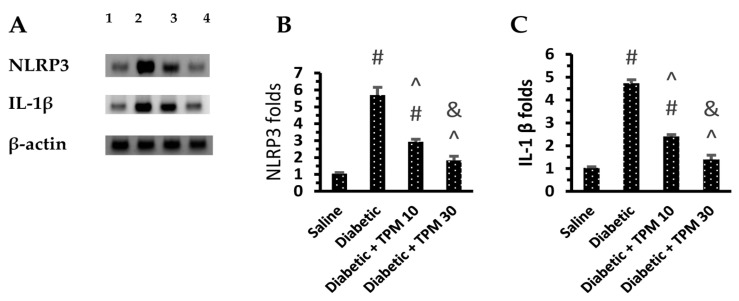
Effect of topiramate on the level of NLRP3 and IL-1β proteins in the retinal homogenates. (**A**) Western blot gels for (1) saline, (2) diabetic, (3 and 4) diabetic + TPM 10 and 30 mg/kg groups. NLRP3: Nod-like receptor family pyrin domain containing 3, IL-1β: interlukin-1β, TPM: topiramate. (**B**,**C**) column charts for NLRP3 and IL-1β. Data in the figures are the means ± SD and analyzed by one-way ANOVA test and post-hoc analysis at *p* < 0.05. #, ^, &: versus saline, diabetic mice and diabetic + TPM group.

Diabetic mice showed high levels of NLRP3, IL-1β, TNF-α, glutamate and MDA (Figure 4A–E) but low GSH versus the saline group (Figure 4F). Treatment with TPM 10 or 30 mg/kg did not suppress the high glutamate level (Figure 4A) but diminished NLRP3, IL-1β, TNF-α and MDA (Figure 4B–E). In addition, TPM 10 or 30 mg/kg restored the retinal GSH level (Figure 4F). In addition, Western blotting indicated 5.7-fold and 4.73-fold increments (Figure 5A) in the retinal NLRP3 and IL-1β levels but reduced dose dependently upon treatment with TPM 10 or 30 mg/kg (Figure 5B,C).

### 3.6. Topiramate Protected Retinal Layers and Improved Total Retinal Thickness

**Figure 6:**
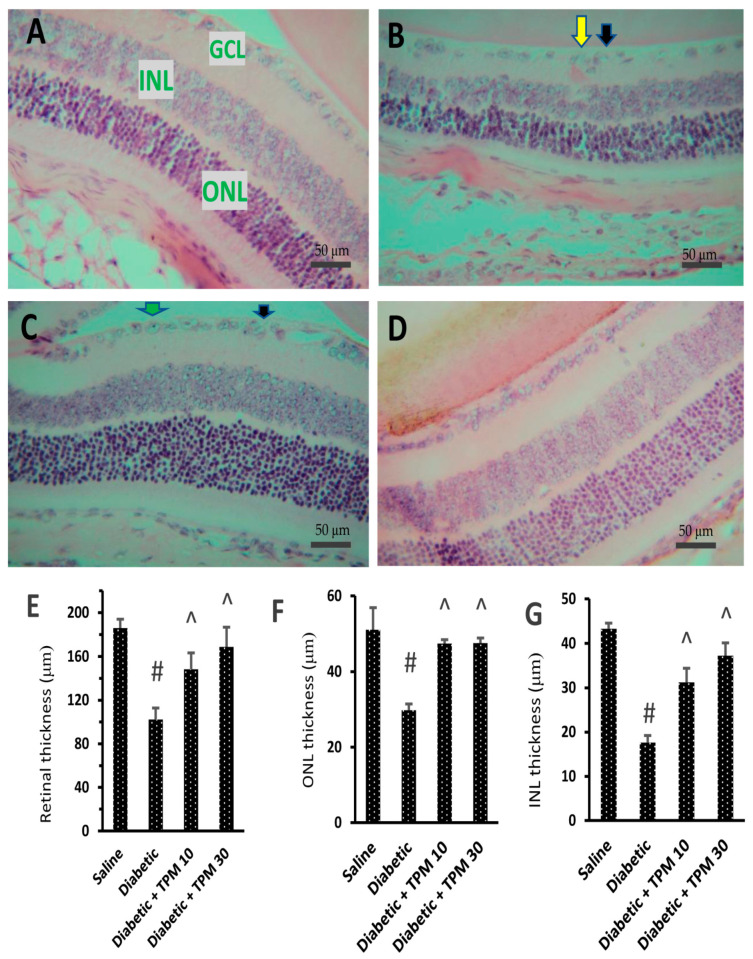
Hematoxylin and eosin staining for retinal sections in the study groups. (**A**) Saline group shows arranged layers in the retina: the ganglionic cell layer (GCL), the inner nuclear layer (INL), and the outer nuclear layer (ONL) (400×). (**B**) The diabetic group shows disorganized GCL (yellow arrow) and some degenerated and vacuolated ganglion cells (black arrows) (×400). (**C**) Diabetic + TPM 10 mg/kg group shows mild vacuolation and few degenerated ganglion cells (black arrows), pyknotic nuclei (green arrow), and ballooning degeneration. (×400). (**D**) Diabetic + TPM 30 mg/kg group shows organized layers and restoration in retinal layer thickness with mild vacuolization (×400) (scale bar 50 µm). TPM: topiramate. (**E**) Mean total retinal thickness in the study groups. (**F**,**G**) Mean ONL and INL thickness. Data in the figures are expressed as mean ± SD. #, ^: versus saline, and diabetic mice, *n* = 5.

In the current study, diabetic mice showed normally structured retinas showing regular thickness of different retinal layers (Figure 6A). However, the diabetic group showed disorganization in the ganglion cell layer (GCL) as well as degenerated and vacuolated ganglion cells (Figure 6B). The diabetic + TPM 10 mg/kg group showed milder vacuolation and fewer degenerated neurons in the GCL, pyknotic nuclei, and ballooning degeneration (Figure 6C). However, the diabetic + TPM 30 mg/kg group showed organized layers and restoration in retinal layer thickness and very mild vacuolization (Figure 6D). The retinal thickness of retinal specimens from the study group is shown in Figure 6E, where the diabetic group showed significantly smaller thickness while the diabetic + TPM 10 or 30 mg/kg groups showed greater thickness. More pathologic features in the diabetic group are shown in Figure S1.

### 3.7. Topiramate Improved the BDNF Level Diabetic Retinas and Optic Nerves

**Figure 7:**
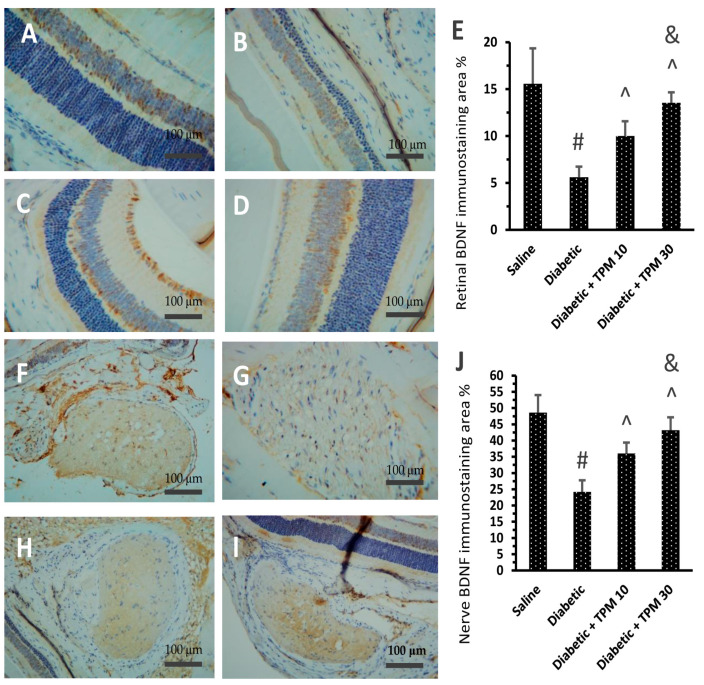
Immunohistochemical staining for BDNF in retinal and optic nerve sections in the experimental groups. (**A**) Saline group: the granular cell layers showed strong and diffuse nuclear positivity. (**B**) Diabetic group: the granular cell layers showed weak and mild nuclear positivity. (**C**) Diabetic + TPM 10 mg/kg group: the granular cell layers showed moderate and focal nuclear positivity. (**D**) Diabetic + TPM 30 mg/kg group: the granular cell layers showed strong and focal nuclear positivity (BDNF X200) (scale bar 100 µm). (**E**) Mean area of BDNF immunostaining. (**F**–**I**) Microscopic images for BDNF immunostaining in the optic nerves in saline, diabetic, diabetic +TPM 10 mg/kg and diabetic + TPM 30 mg/kg groups (BDNF ×200) (scale bar 100 µm). (**J**) Mean area of BDNF immunostaining. TPM: topiramate, BDNF: brain-derived neurotrophic factor. Data in the figures are expressed as mean ± SD. #, ^, &: versus saline, diabetic mice and diabetic + TPM group at *p* < 0.05, *n* = 5.

In the current study, immunostaining for BDNF in the retinas was different among the study groups. The saline group showed moderate staining for BDNF in the retinas (Figure 7A), whereas the diabetic group showed the lowest staining intensity (Figure 7B). The diabetic + TPM 10 m/kg group showed mild–moderate staining (Figure 7C), but BDNF staining was improved in the diabetic + TPM 30 mg/kg group (Figure 7D). Figure 7E demonstrates the area of BDNF immunostaining in the study groups, and dose-dependent significant improvements were detected in diabetic + TPM 10 and 30 mg/kg groups. Similar results were obtained upon quantification of immunostaining for BDNF in the optic nerves. The saline group showed moderate staining for BDNF (Figure 7F) whereas the diabetic group showed weak staining (Figure 7G). The diabetic + TPM 10 m/kg group showed mild–moderate staining (Figure 7H), but BDNF staining was improved in the diabetic + TPM 30 mg/kg group (Figure 7I). Panel 8E demonstrates the area of BDNF immunostaining in the optic nerves, and dose-dependent significant improvements were detected in diabetic + TPM 10 and 30 mg/kg groups.

### 3.8. Topiramate Protected against Ultrastructural Pathology in Diabetic Retinas

**Figure 8:**
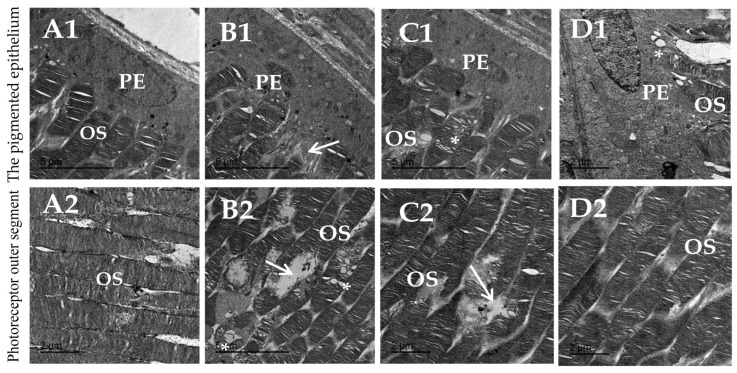
Micrographs taken by the transmission electron for the pigmented epithelium layer and photoreceptor outer segment. (**A1**,**A2**) Images from the saline group show normal pigmented epithelium (PE) shape having normal arranged inner stacked membrane of outer segment. (**B1**,**B2**) Diabetes group showing degenerated nucleus of pigmented epithelium, dissolution of photoreceptor stacked membranes (arrow), with multi-vacuolation in outer segments (*) in between. (**C1**,**C2**) Diabetic + TPM 10 mg/kg group shows decreasing vacuolation (*) in outer segments and still degeneration (arrow) of stacked membrane of outer segments (**D1**,**D2**). Diabetic + TPM 10 mg/kg group shows normal pigmented epithelia with normal nucleus shape arrangement of outer segments, noticed improvement of its stacked membrane with few vacuoles (*).

**Figure 9:**
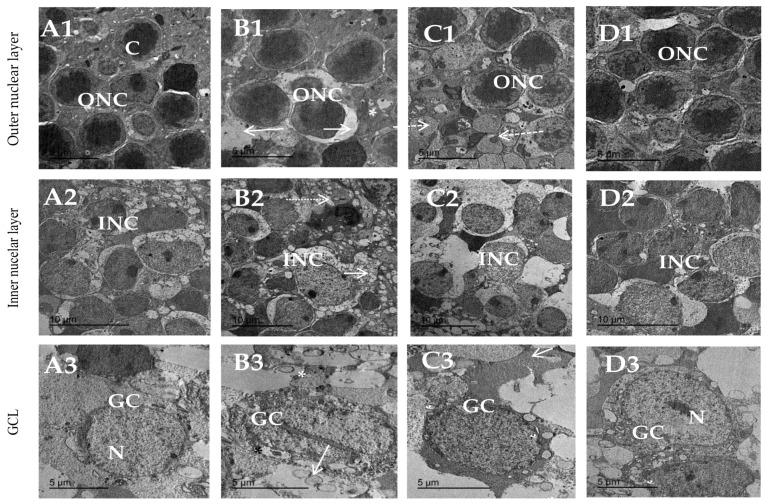
Transmission electron micrograph of outer nuclear layer, inner nuclear layer and GCL. (**A1**) Saline group show normal pattern of outer nuclear cells with normal highly condensed centrally located chromatin (C). (**B1**) Diabetes group showing different spots of harmed cells of outer nuclear layer (arrow) and swallow cells with abnormal distribution of chromatin material and few vacuoles (*). (**C1**) Diabetic + TPM 10 mg/kg group showing slight amelioration in outer nuclear cells shape with still degenerated cells (dotted arrow). (**D1**) Diabetic + TPM 30 mg/kg group showing normal outer nuclear ultrastructure shape with slightly round shape nucleus as normal. (**A2**) Saline group of inner nuclear cells (INC) showing normal distribution and normal shape, and bipolar cells of inner nuclear layer enclosed in between Muller cells. (**B2**) Diabetes group showing massive inner nuclear cell damage; most of cells show pleomorphic nuclei (*) and some necrotic cells (arrow) with multi-vacuolation (dotted arrow). (**C2**) Diabetic + TPM 10 mg/kg group showing inner nuclear layer cells restored their normal size and shape and few vacuolations (*). (**D2**) Diabetic + TPM 30 mg/kg group showing nearly normal distribution of inner nuclear cells forming the typical shape of this layer with normal intact cells. (**A3**) Saline group showing normal cell shape with normal round nucleus. (**B3**) Diabetes group showing damaged ganglion cells with multiple vacuolations (*) and ruptured cell membranes (arrow). (**C3**) Diabetic + TPM 10 mg/kg group shows few vacuoles and normal outline cell shape. (**D3**) Diabetic + TPM 30 mg/kg group shows ganglion cells with regular structure and smaller vacuolation. TPM: topiramate, GCL: ganglion cell layer.

The ultrastructural pathologies in the retinas are shown in Figure 8 and Figure 9. Figure 8A1,A2 show a normal arrangement of the inner stacked membrane of the outer segment. Figure 8B1,B2 show degenerated nuclei of pigmented epithelium and dissolution of photoreceptor stacked membranes in the diabetic group. Figure 8C1,C2 from the diabetic + TPM 10 mg/kg group showed decreased vacuolation in outer segments and still degeneration of stacked membrane of outer segments. Figure 8D1,D2 from the diabetic + TPM 30 mg/kg group show nearly normal pigmented epithelium with normal nucleus shape arrangement of outer segments. 

Figure 9 shows transmission electron micrographs for the outer nuclear layer, inner nuclear layer and GCL of different experimental groups. Regarding the outer nuclear layer, the saline group (Figure 9A1) shows a normal pattern of cells, whereas the diabetic group (Figure 9B1) shows different spots of harmed cells of the outer nuclear layer. The diabetic + TPM 10 mg/kg group shows slight amelioration in outer nuclear cell shape (Figure 9C1), while the diabetic + TPM 30 mg/kg group shows a normal outer nuclear ultrastructure shape with a slightly round shape nucleus (Figure 9D1). Regarding the inner nuclear layer, the saline group showed normal distribution and normal shape, and bipolar cells of the inner nuclear layer enclosed in between Muller cells (Figure 9A2). The diabetic group showed massive inner nuclear cell damage (Figure 9B2). The diabetic + TPM 10 mg/kg group showed inner nuclear layer cells with typical size and shape and few vacuolations (Figure 9C2). The diabetic + TPM 30 mg/kg group showed a regular distribution of cells of the INL (Figure 9D2). Regarding the GCL, the saline group showed a normal cell shape with a normal round nucleus (Figure 9A3), whereas the diabetic group showed damaged ganglion cells (Figure 9B3). The diabetic + TPM 10 mg/kg group showed few vacuoles and a normal outline (Figure 9C3). The diabetic + TPM 30 mg/kg group showed ganglion cells with normal structures and smaller vacuolations (Figure 9D3). TPM: topiramate, GCL: ganglion cell layer, INL: inner nuclear layer.

## 4. Discussion

The antiepileptic drug topiramate has antioxidant and anti-inflammatory effects in inflammatory conditions. In addition, it can improve glycemic control in experimental rats and type 2 DM patients [34,35]. In this study, the computational study determined the potential target prediction of topiramate’s role in the treatment of DREN. Further, genes/proteins targeted in DREN were identified; the combined analysis revealed 118 unique targets: those represented the input for topiramate in the drug–disease analysis. Surprisingly, they were highly localized in neuronal cells, ion channels and complexes. A Venn diagram showed 27 overlapped genes between topiramate and DREN. Analysis of the cellular component revealed the involvement of NF-κB and the inflammasome. These data offered a rationale for designing the experimental study.

To our knowledge, the protective effect of topiramate against DREN and its molecular mechanisms in relation to NLRP3 inflammasome has not been elucidated yet. Moreover, the effect of topiramate on BDNF remains to be determined. In the present study, we aimed to investigate the possible protective effect of topiramate on DREN in mice and explore its underlying mechanisms, especially those associated with NLRP3/IL-1β signaling, OXS and BDNF expression. 

Pathology of DREN involves various ROS-producing processes, such as glucose auto-oxidation, non-controlled endoplasmic reticulum stress, activation of the polyol pathway, protein glycation and production of complex glycation end-products that activate the signaling of the NLRP3 inflammasome pathway [36]. Indeed, increased MDA and decreased GSH levels demonstrated the dysregulation of oxidant/antioxidant capacity [37,38,39].

In the current study, histopathological examination confirmed the development of DREN by detecting vacuolar degeneration and disorganization of most ganglion cells. However, topiramate treatment ameliorated the chronic hyperglycemia-induced histological alterations with more pronounced effects observed with the 30 mg/kg dose than with the 10 mg/kg dose. In agreement with this, Yoneda et al. [40] indicated that the systemic treatment with topiramate prevented ischemia-induced histopathological and functional disruption in rat retina in a dose-dependent manner. Another mechanism of retinal neurodegeneration by glutamate excitotoxicity is through nitric oxide [41]. It enhances the fragmentation of RGCs mitochondria, resulting in the upregulation of the NMDA receptor, increased OXS and the initiation of neurodegeneration.

In diabetic mice, our results showed elevated levels of retinal glutamate, MDA, NLRP3, TNF-α and IL-1β but reduced GSH. Inflammation mediates a crucial pathogenic role in DREN. Low-grade inflammation has been identified extensively in diabetic patients and animal studies. Inflammasomes, especially NLRP3, play a vital role in innate immunity by stimulating caspase- 1, facilitating the production of IL-1β and the release of mature cytokines. Additionally, inflammasomes control cell inflammatory necrosis (pyroptosis), which triggers cellular inflammation during pathological inflammatory or stressful conditions. The NLRP3 inflammasome is considered one of the most investigated members of the NLR family. NLRP3 inflammasomes were demonstrated to have essential roles in DREN pathogenesis [42,43]. 

Various complications of DM, such as DREN, may develop as a result of complex interaction between inflammatory and metabolic changes [44]. The NLRP3 inflammasome is thought to be activated during this process [45]. When stimulated, the NLRP3 protein clumps with the apoptosis-associated speck-like protein comprised of a caspase-recruitment domain (ASC) and procaspase-1. In turn, this activates procaspase-1 and releases the active form of IL-1β and IL-18 that mediates the downstream inflammation cascade in DREN [46]. In line with previous reports, our results indicated that the overexpression of key NLRP3 inflammasomes is evident in diabetic retinas, and the activation of NLRP3 inflammasomes led to the upregulation of the inflammatory cytokines, TNF-α and IL-1β.

Chronic hyperglycemia activates transcriptional factors that upregulate pro-inflammatory molecules, causing low-grade inflammation in the retina [47]. This low-grade inflammatory state increases the expression of intercellular adhesion molecule (ICAM)-1, which leads to the release of cytokines, chemokines and proinflammatory and pro-angiogenic growth factors in the retina [48]. Furthermore, the blood–retinal barrier is broken down by these inflammatory mediators that lead to the development of intraretinal edema [49]. Low-grade proinflammatory activity causes damage to pericytes around endothelial cells and the weakening of endothelial walls, which triggers the progress of vascular abnormalities [50].

On the other hand, topiramate significantly lowered retinal NLRP3, TNF-α, IL-1β and MDA and significantly increased retinal GSH in the treated diabetic group. In addition, higher topiramate doses produced more significant improvement. Similarly, the antioxidant and anti-inflammatory effects of topiramate in other tissues were observed. In agreement, topiramate was reported to decrease lipopolysaccharide-stimulated IL-1β and IL-6 from rat microglial cells in a dose-dependent manner [51]. Moreover, topiramate reduced lipid peroxidation, IL-1β and TNF-α levels; increased the previously reduced glutathione levels and exerted neuroprotective effects against methylphenidate-induced neurodegeneration and spinal cord injury [52,53]. It also protected against indomethacin-induced gastric ulcers by inhibiting OXS in gastric tissue [54]. Nevertheless, to our best knowledge, this is the first study to examine the effect of topiramate on NLRP3. 

Furthermore, excitotoxicity is a pathologic phenomenon in which overstimulation of glutamate receptors results in damaged neurons [55]. A large body of research suggests that the vitreous and retina of both experimental animal models of diabetes and diabetic patients have higher glutamate levels [56,57]. The impaired function of the glutamate transporters on retinal Muller cells and, possibly, reduced expression of glutamine synthase are suggested to be contributing factors to the higher glutamate levels [56]. Under physiological conditions, light stimulation leads to the release of glutamate from the retinal photoreceptors, bipolar cells and ganglion cells, leading to activation of NMDA receptors. When NMDA are activated, depolarization of neuronal cells increases with the influx of Ca^2+^ and Na^+^ into the cell; however, prolonged exposure of neurons to glutamate causes cellular death by increasing the intracellular Ca2^+^, which, in turn, generates free radicals and induces neuronal cell apoptosis [58]. Neuronal death, particularly retinal GCL death, occurs in multiple retinal diseases, including DREN [59]. The protective effect of topiramate on excitotoxic cell death has been previously highlighted. Topiramate is an AMPA-specific glutamate receptor antagonist. Besides its antagonizing effect, it inhibits the activity of voltage-gated Na^+^ and Ca^2+^ channels and blocks the influx of calcium into cells [60,61].

Immunostaining of the diabetic mice retina in our study displayed a decline in the retinal BDNF level; a similar decline in DBNF was recorded in the retinas of diabetic rodents [62] and the serum of DREN patients [63]. BDNF deficiency in DREN contributes to an aberrant increase in autophagy via enhancing the expression of microtubule-associated protein light chain 3 B (LC3B) (autophagosome involved in the degradation of gap junction connexin leading to vascular cell death in the retina) [64]. BDNF is a neuroprotective factor that may halt the pathogenesis and progression of DREN. BDNF provides a retinal neuroprotective effect; it can suppress apoptosis and promote RGC regeneration in the retina. Low BDNF levels are linked to retinal neovascularization in DREN patients. In the early stages of DREN, it is thought that BDNF has its optimal concentration and best neuroprotective effect [65].

Interestingly, topiramate promoted BDNF expression in the retinas of diabetic-treated mice. In addition, a more pronounced effect was observed with the higher dose of topiramate. Indeed, topiramate treatment increased the BDNF gene in methylphenidate-induced neurodegeneration. Topiramate interacts with AMPA/kainate, GABAA, NMDA, and α2-adrenergic receptors to improve BDNF expression and provides neuroprotection against methylphenidate-induced neurodegeneration [52]. Studies have reported the lack of a direct effect of topiramate on NMDA receptors. However, the activity of NMDA receptor subtypes was reported to be influenced by the activity of the AMPA receptor. Hence, it is probable that topiramate has the ability to affect NMDA-induced actions [66]. The binding of topiramate to vacant phosphorylation locations within one or more proteins that contain the AMPA or kainate receptor complexes prevents phosphorylation and may show allosteric modulatory action on channel activity. By this mechanism, topiramate could mediate NMDA receptor activity through its action on AMPA receptors [60].

We have a justification for the schedule of topiramate dosing. A typical human treatment dose of topiramate is 400 mg per day and can be given once or divided into two doses. In the present study, two doses of topiramate (10 and 30 mg/kg) were used. We can translate the mouse dose to the human equivalent by applying the Reagan–Shaw method [67]. Using the formula: the human equivalent dose (in mg/kg) equals the animal dose (in mg/kg) × animal (km)/human (km). The Km is a fixed factor for a 60 kg human adult equals 37, and the Km for a 20 g mouse equals 3. Thus, the human equivalent of a murine dose of 10 and 30 mg/kg are 48.6 and 145.8 mg for an average-sized, 60 kg adult human. Therefore, all the selected doses in the present study are within the safe therapeutic range recorded in humans.

Several studies documented the role of antioxidants in clinical and experimental models of epilepsy and other disorders. Previous studies reported similar antioxidant and anti-inflammatory actions for topiramate. One study provided evidence that topiramate provides free radical scavenging activity in vitro in a dose-dependent manner [20]. Kubera et al. (2004) studied the impact of intraperitoneal topiramate (40 and 80 mg/kg) on the fully developed kainate (15 mg/kg)-induced status epilepticus in rats and found lessened lipid peroxidation in the cortex [68]. Another group investigated the effect of topiramate and/or selenium on reduced lipoperoxidation in the cortex of pentylenetetrazole-intoxicated rats [69]. Another study indicated that topiramate and vitamin E treatment caused a decline in plasma lipoperoxidation levels and brain spike numbers, while topiramate produced increments in GSH-Px, GSH and latency to the first spike of EEG [70]. 

Topiramate pretreatment also reduced the contents of tissue malonaldehyde, enhanced glutathione levels and increased the activity of superoxide dismutase, catalase and glutathione peroxidase in gastric mucosa of rats subjected to indomethacin-induced peptic ulcers [54]. Another research group showed recovery and enhanced immunoreactivities and protein levels of antioxidant enzymes in the mice hippocampal dentate gyrus subjected to D-galactose-induced neuroblast injury [71]. However, when topiramate is used as an antiepileptic or migraine drug, it was not observed in patients concurrently affected by diabetes whether it protects from DREN as well or not.

Together, our data suggested that topiramate may be a new therapeutic option for DREN. The underlying mechanisms involve downregulating OXS and reducing NLRP3/IL-1β signaling, which further decreases the inflammatory burden and promotes BDNF production. Hence, topiramate neuroprotective in DREN and future studies is warranted to further explore its protective mechanism and possible influence on retinal angiogenesis and endothelial protection.

## Data Availability

Data are available from the authors upon request.

## Supplementary Materials

The following supporting information can be downloaded at: https://www.mdpi.com/article/10.3390/biomedicines11123202/s1. Supplementary Figure S1. Image shows disorganized ganglion cell layer and vacuolation (panel A) and pyknotic nuclei (panel B) at high power (400×).

## References

[1] Wong T.Y., Cheung C.M.G., Larsen M., Sharma S., Simó R. (2016). Diabetic Retinopathy. Nat. Rev. Dis. Primers.

[2] Mahajan N., Arora P., Sandhir R. (2019). Perturbed Biochemical Pathways and Associated Oxidative Stress Lead to Vascular Dysfunctions in Diabetic Retinopathy. Oxidative Med. Cell. Longev..

[3] Liu K., Gao X., Hu C., Gui Y., Gui S., Ni Q., Tao L., Jiang Z. (2022). Capsaicin Ameliorates Diabetic Retinopathy by Inhibiting Poldip2-Induced Oxidative Stress. Redox Biol..

[4] Price T.O., Eranki V., Banks W.A., Ercal N., Shah G.N. (2012). Topiramate Treatment Protects Blood-Brain Barrier Pericytes from Hyperglycemia-Induced Oxidative Damage in Diabetic Mice. Endocrinology.

[5] Liu C., Dong W., Lv Z., Kong L., Ren X. (2022). Thioredoxin-Interacting Protein in Diabetic Retinal Neurodegeneration: A Novel Potential Therapeutic Target for Diabetic Retinopathy. Front. Neurosci..

[6] Yu X., Xu Z., Mi M., Xu H., Zhu J., Wei N., Chen K., Zhang Q., Zeng K., Wang J. (2008). Dietary Taurine Supplementation Ameliorates Diabetic Retinopathy via Anti-Excitotoxicity of Glutamate in Streptozotocin-Induced Sprague-Dawley Rats. Neurochem. Res..

[7] Zhang X., Dai J., Li L., Chen H., Chai Y. (2017). NLRP3 Inflammasome Expression and Signaling in Human Diabetic Wounds and in High Glucose Induced Macrophages. J. Diabetes Res..

[8] Sun Y., Ding S. (2021). NLRP3 Inflammasome in Diabetic Cardiomyopathy and Exercise Intervention. Int. J. Mol. Sci..

[9] Murakami T., Ockinger J., Yu J., Byles V., McColl A., Hofer A.M., Horng T. (2012). Critical Role for Calcium Mobilization in Activation of the NLRP3 Inflammasome. Proc. Natl. Acad. Sci. USA..

[10] Weber K., Schilling J.D. (2014). Lysosomes Integrate Metabolic-Inflammatory Cross-Talk in Primary Macrophage Inflammasome Activation. J. Biol. Chem..

[11] Kelley N., Jeltema D., Duan Y., He Y. (2019). The NLRP3 Inflammasome: An Overview of Mechanisms of Activation and Regulation. Int. J. Mol. Sci..

[12] Muñoz-Planillo R., Kuffa P., Martínez-Colón G., Smith B.L., Rajendiran T.M., Núñez G. (2013). K^+^ Efflux Is the Common Trigger of NLRP3 Inflammasome Activation by Bacterial Toxins and Particulate Matter. Immunity.

[13] Cheung N., Wong I.Y., Wong T.Y. (2014). Ocular Anti-VEGF Therapy for Diabetic Retinopathy: Overview of Clinical Efficacy and Evolving Applications. Diabetes Care.

[14] Fogli S., Del Re M., Rofi E., Posarelli C., Figus M., Danesi R. (2018). Clinical Pharmacology of Intravitreal Anti-VEGF Drugs. Eye.

[15] Santhakumaran S., Salimi A., Brunetti V., Galic J. (2022). Efficacy and Safety of Aflibercept Therapy for Diabetic Macular Edema: A Systematic Review and Meta-Analysis. J. Curr. Ophthalmol..

[16] Kiire C.A., Morjaria R., Rudenko A., Fantato A., Smith L., Smith A., Chong V. (2015). Intravitreal Pegaptanib for the Treatment of Ischemic Diabetic Macular Edema. Clin. Ophthalmol..

[17] Devi T.S., Yumnamcha T., Yao F., Somayajulu M., Kowluru R.A., Singh L.P. (2019). TXNIP Mediates High Glucose-Induced Mitophagic Flux and Lysosome Enlargement in Human Retinal Pigment Epithelial Cells. Biol. Open.

[18] Li X., Kover K.L., Heruth D.P., Watkins D.J., Guo Y., Moore W.V., He L.G., Zang M., Clements M.A., Yan Y. (2017). Thioredoxin-Interacting Protein Promotes High-Glucose-Induced Macrovascular Endothelial Dysfunction. Biochem. Biophys. Res. Commun..

[19] Abo-Elmatty D.M., Zaitone S.A. (2011). Topiramate Induces Weight Loss and Improves Insulin Sensitivity in Dietary Obese Rats: Comparison to Sibutramine. Eur. Rev. Med. Pharmacol. Sci..

[20] Cárdenas-Rodríguez N., Coballase-Urrutia E., Rivera-Espinosa L., Romero-Toledo A., Sampieri A., Ortega-Cuellar D., Montesinos-Correa H., Floriano-Sánchez E., Carmona-Aparicio L. (2013). Modulation of Antioxidant Enzymatic Activities by Certain Antiepileptic Drugs (Valproic Acid, Oxcarbazepine, and Topiramate): Evidence in Humans and Experimental Models. Oxid. Med. Cell. Longev..

[21] Cárdenas-Rodríguez N., Coballase-Urrutia E., Huerta-Gertrudis B., García-Cruz M.E., Pedraza-Chaverri J., Coria-Jiménez R., Bandala C., Ruíz-García M. (2013). Antioxidant Activity of Topiramate: An Antiepileptic Agent. Neurol. Sci..

[22] Kikumoto Y., Sugiyama H., Inoue T., Morinaga H., Takiue K., Kitagawa M., Fukuoka N., Saeki M., Maeshima Y., Wang D.-H. (2010). Sensitization to Alloxan-Induced Diabetes and Pancreatic Cell Apoptosis in Acatalasemic Mice. Biochim. Biophys. Acta Mol. Basis Dis..

[23] Elsherbiny N.M., Abdel-Mottaleb Y., Elkazaz A.Y., Atef H., Lashine R.M., Youssef A.M., Ezzat W., El-Ghaiesh S.H., Elshaer R.E., El-Shafey M. (2019). Carbamazepine Alleviates Retinal and Optic Nerve Neural Degeneration in Diabetic Mice via Nerve Growth Factor-Induced PI3K/Akt/mTOR Activation. Front. Neurosci..

[24] Agarwal N.B., Agarwal N.K., Mediratta P.K., Sharma K.K. (2011). Effect of Lamotrigine, Oxcarbazepine and Topiramate on Cognitive Functions and Oxidative Stress in PTZ-Kindled Mice. Seizure.

[25] Liang Y., Chen X., Osborne M., DeCarlo S.O., Jetton T.L., Demarest K. (2005). Topiramate Ameliorates Hyperglycaemia and Improves Glucose-Stimulated Insulin Release in ZDF Rats and Db/Db Mice. Diabetes Obes. Metab..

[26] Attia M.A., Soliman N., Eladl M.A., Bilasy S.E., El-Abaseri T.B., Ali H.S., Abbas F., Ibrahim D., Osman N.M.S., Hashish A.A. (2023). Topiramate Affords Neuroprotection in Diabetic Neuropathy Model via Downregulating Spinal GFAP/Inflammatory Burden and Improving Neurofilament Production. Toxicol. Mech. Methods.

[27] Bari M.W., Islam M.M., Khatun M., Sultana M.J., Ahmed R., Islam A., Hossain M.I., Rahman M.M., Islam M.A. (2020). Antidiabetic Effect of Wedelia Chinensis Leaf Extract in Alloxan Induced Swiss Albino Diabetic Mice. Clin. Phytosci..

[28] Halliwell B. (1992). Reactive Oxygen Species and the Central Nervous System. J. Neurochem..

[29] Ohkawa H., Ohishi N., Yagi K. (1979). Assay for Lipid Peroxides in Animal Tissues by Thiobarbituric Acid Reaction. Anal. Biochem..

[30] Beutler E., Duron O., Kelly B.M. (1963). Improved Method for the Determination of Blood Glutathione. J. Lab. Clin. Med..

[31] Jo Y.-J., Sonoda K.-H., Oshima Y., Takeda A., Kohno R., Yamada J., Hamuro J., Yang Y., Notomi S., Hisatomi T. (2011). Establishment of a New Animal Model of Focal Subretinal Fibrosis That Resembles Disciform Lesion in Advanced Age-Related Macular Degeneration. Investig. Ophthalmol. Vis. Sci..

[32] Hayat M.A. (1989). Principles and Techniques of Electron Microscopy: Biological Applications.

[33] Hermenean A., Trotta M.C., Gharbia S., Hermenean A.G., Peteu V.E., Balta C., Cotoraci C., Gesualdo C., Rossi S., Gherghiceanu M. (2021). Changes in Retinal Structure and Ultrastructure in the Aged Mice Correlate With Differences in the Expression of Selected Retinal miRNAs. Front. Pharmacol..

[34] Toplak H., Hamann A., Moore R., Masson E., Gorska M., Vercruysse F., Sun X., Fitchet M., for the OBDM-002 Study Group (2007). Efficacy and Safety of Topiramate in Combination with Metformin in the Treatment of Obese Subjects with Type 2 Diabetes: A Randomized, Double-Blind, Placebo-Controlled Study. Int. J. Obes..

[35] Shafik A.N. (2012). Effects of Topiramate on Diabetes Mellitus Induced by Streptozotocin in Rats. Eur. J. Pharmacol..

[36] Menu P., Mayor A., Zhou R., Tardivel A., Ichijo H., Mori K., Tschopp J. (2012). ER Stress Activates the NLRP3 Inflammasome via an UPR-Independent Pathway. Cell Death Dis..

[37] Ali S.A., Zaitone S.A., Dessouki A.A., Ali A.A. (2019). Pregabalin Affords Retinal Neuroprotection in Diabetic Rats: Suppression of Retinal Glutamate, Microglia Cell Expression and Apoptotic Cell Death. Exp. Eye Res..

[38] Zaitone S.A., Alshaman R., Alattar A., Elsherbiny N.M., Abogresha N.M., El-Kherbetawy M.K., Elaskary A.A., Hashish A.A., Rashed L.A., Ahmed E. (2020). Retinoprotective Effect of Donepezil in Diabetic Mice Involves Mitigation of Excitotoxicity and Activation of PI3K/mTOR/BCl_2_ Pathway. Life Sci..

[39] ElSayed M.H., Elbayoumi K.S., Eladl M.A., Mohamed A.A.K., Hegazy A., El-Sherbeeny N.A., Attia M.A., Hisham F.A., Saleh M.A.K., Elaskary A. (2023). Memantine Mitigates ROS/TXNIP/NLRP3 Signaling and Protects against Mouse Diabetic Retinopathy: Histopathologic, Ultrastructural and Bioinformatic Studies. Biomed. Pharmacother..

[40] Yoneda S., Tanaka E., Goto W., Ota T., Hara H. (2003). Topiramate Reduces Excitotoxic and Ischemic Injury in the Rat Retina. Brain Res..

[41] Nguyen D., Alavi M.V., Kim K.-Y., Kang T., Scott R.T., Noh Y.H., Lindsey J.D., Wissinger B., Ellisman M.H., Weinreb R.N. (2011). A New Vicious Cycle Involving Glutamate Excitotoxicity, Oxidative Stress and Mitochondrial Dynamics. Cell Death Dis..

[42] Devi T.S., Lee I., Hüttemann M., Kumar A., Nantwi K.D., Singh L.P. (2012). TXNIP Links Innate Host Defense Mechanisms to Oxidative Stress and Inflammation in Retinal Muller Glia under Chronic Hyperglycemia: Implications for Diabetic Retinopathy. Exp. Diabetes Res..

[43] Chen W., Zhao M., Zhao S., Lu Q., Ni L., Zou C., Lu L., Xu X., Guan H., Zheng Z. (2017). Activation of the TXNIP/NLRP3 Inflammasome Pathway Contributes to Inflammation in Diabetic Retinopathy: A Novel Inhibitory Effect of Minocycline. Inflamm. Res..

[44] Pan W.W., Lin F., Fort P.E. (2021). The Innate Immune System in Diabetic Retinopathy. Prog. Retin. Eye Res..

[45] Raman K.S., Matsubara J.A. (2022). Dysregulation of the NLRP3 Inflammasome in Diabetic Retinopathy and Potential Therapeutic Targets. Ocul. Immunol. Inflamm..

[46] Forrester J.V., Kuffova L., Delibegovic M. (2020). The Role of Inflammation in Diabetic Retinopathy. Front. Immunol..

[47] Tang J., Kern T.S. (2011). Inflammation in Diabetic Retinopathy. Prog. Retin. Eye Res..

[48] Rübsam A., Parikh S., Fort P. (2018). Role of Inflammation in Diabetic Retinopathy. Int. J. Mol. Sci..

[49] Abu El-Asrar A. (2012). Role of Inflammation in the Pathogenesis of Diabetic Retinopathy. Middle East Afr. J. Ophthalmol..

[50] dell’Omo R., Semeraro F., Bamonte G., Cifariello F., Romano M.R., Costagliola C. (2013). Vitreous Mediators in Retinal Hypoxic Diseases. Mediat. Inflamm..

[51] Andrzejczak D., Woldan-Tambor A., Bednarska K., Zawilska J.B. (2016). The Effects of Topiramate on Lipopolysaccharide (LPS)-Induced Proinflammatory Cytokine Release from Primary Rat Microglial Cell Cultures. Epilepsy Res..

[52] Motaghinejad M., Motevalian M., Fatima S. (2017). Mediatory Role of NMDA, AMPA/Kainate, GABA A and Alpha 2 Receptors in Topiramate Neuroprotective Effects against Methylphenidate Induced Neurotoxicity in Rat. Life Sci..

[53] Narin F., Hanalioglu S., Ustun H., Kilinc K., Bilginer B. (2017). Topiramate as a Neuroprotective Agent in a Rat Model of Spinal Cord Injury. Neural Regen. Res..

[54] Jafari A., Andishfar N., Esmaeilzadeh Z., Khezri M.R., Ghasemnejad-Berenji M. (2022). Gastroprotective Effect of Topiramate on Indomethacin-induced Peptic Ulcer in Rats: Biochemical and Histological Analyses. Basic Clin. Pharmacol. Toxicol..

[55] Wal P., Dwivedi J., Wal A., Vig H., Singh Y. (2022). Detailed Insight into the Pathophysiology and the Behavioral Complications Associated with the Parkinson’s Disease and Its Medications. Future J. Pharm. Sci..

[56] Ishikawa M. (2013). Abnormalities in Glutamate Metabolism and Excitotoxicity in the Retinal Diseases. Scientifica.

[57] Ola M.S., Alhomida A.S., LaNoue K.F. (2019). Gabapentin Attenuates Oxidative Stress and Apoptosis in the Diabetic Rat Retina. Neurotox. Res..

[58] Ola M., Nawaz M., Khan H., Alhomida A. (2013). Neurodegeneration and Neuroprotection in Diabetic Retinopathy. Int. J. Mol. Sci..

[59] Whitmire W., Al-Gayyar M.M., Abdelsaid M., Yousufzai B.K., El-Remessy A.B. (2011). Alteration of Growth Factors and Neuronal Death in Diabetic Retinopathy: What We Have Learned so Far. Mol. Vis..

[60] Shank R.P., Maryanoff B.E. (2008). Molecular Pharmacodynamics, Clinical Therapeutics, and Pharmacokinetics of Topiramate. CNS Neurosci. Ther..

[61] Gensel J.C., Tovar C.A., Bresnahan J.C., Beattie M.S. (2012). Topiramate Treatment Is Neuroprotective and Reduces Oligodendrocyte Loss after Cervical Spinal Cord Injury. PLoS ONE.

[62] Platania C.B.M., Maisto R., Trotta M.C., D’Amico M., Rossi S., Gesualdo C., D’Amico G., Balta C., Herman H., Hermenean A. (2019). Retinal and Circulating Mi RNA Expression Patterns in Diabetic Retinopathy: An in Silico and in Vivo Approach. Br. J. Pharmacol..

[63] Jun Y.H., Kim S.T. (2021). Brain-Derived Neurotrophic Factor in Non-Proliferative Diabetic Retinopathy with Diabetic Macular Edema. Eur. J. Ophthalmol..

[64] Trotta M.C., Maisto R., Guida F., Boccella S., Luongo L., Balta C., D’Amico G., Herman H., Hermenean A., Bucolo C. (2019). The Activation of Retinal HCA2 Receptors by Systemic Beta-Hydroxybutyrate Inhibits Diabetic Retinal Damage through Reduction of Endoplasmic Reticulum Stress and the NLRP3 Inflammasome. PLoS ONE.

[65] Afarid M., Namvar E., Sanie-Jahromi F. (2020). Diabetic Retinopathy and BDNF: A Review on Its Molecular Basis and Clinical Applications. J. Ophthalmol..

[66] Du J., Creson T.K., Wu L.-J., Ren M., Gray N.A., Falke C., Wei Y., Wang Y., Blumenthal R., Machado-Vieira R. (2008). The Role of Hippocampal GluR1 and GluR2 Receptors in Manic-Like Behavior. J. Neurosci..

[67] Reagan-Shaw S., Nihal M., Ahmad N. (2008). Dose Translation from Animal to Human Studies Revisited. FASEB J..

[68] Kubera M., Budziszewska B., Jaworska-Feil L., Basta-Kaim A., Leśkiewicz M., Tetich M., Maes M., Kenis G., Marciniak A., Czuczwar S.J. (2004). Effect of Topiramate on the Kainate-Induced Status Epilepticus, Lipid Peroxidation and Immunoreactivity of Rats. Pol. J. Pharmacol..

[69] Nazıroğlu M., Kutluhan S., Yılmaz M. (2008). Selenium and Topiramate Modulates Brain Microsomal Oxidative Stress Values, Ca2+-ATPase Activity, and EEG Records in Pentylentetrazol-Induced Seizures in Rats. J. Membr. Biol..

[70] Nazıroğlu M., Kutluhan S., Uğuz A.C., Çelik Ö., Bal R., Butterworth P.J. (2009). Topiramate and Vitamin E Modulate the Electroencephalographic Records, Brain Microsomal and Blood Antioxidant Redox System in Pentylentetrazol-Induced Seizure of Rats. J. Membr. Biol..

[71] Shen H., Wang J., Jiang D., Xu P., Zhu X., Zhang Y., Yu X., Won M.-H., Su P.Q., Yan B.C. (2017). Topiramate Improves Neuroblast Differentiation of Hippocampal Dentate Gyrus in the D-Galactose-Induced Aging Mice via Its Antioxidant Effects. Cell Mol. Neurobiol..

