# Supplementary material for: Protective Effect of Topiramate against Diabetic Retinopathy and Computational Approach Recognizing the Role of NLRP3/IL-1β/TNF-α Signaling
Source: Biomedicines. 2023 Dec 1;11(12):3202. doi: 10.3390/biomedicines11123202 (PMC10741203; doi:10.3390/biomedicines11123202)
Supplement: Supplementary file 1 [file biomedicines-11-03202-s001.zip › biomedicines-2603343-supplementary.pdf]

A	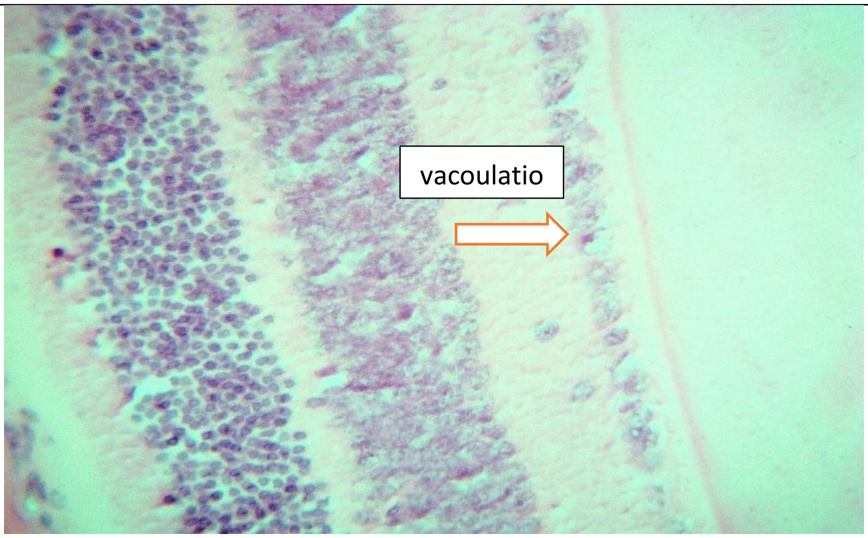
B	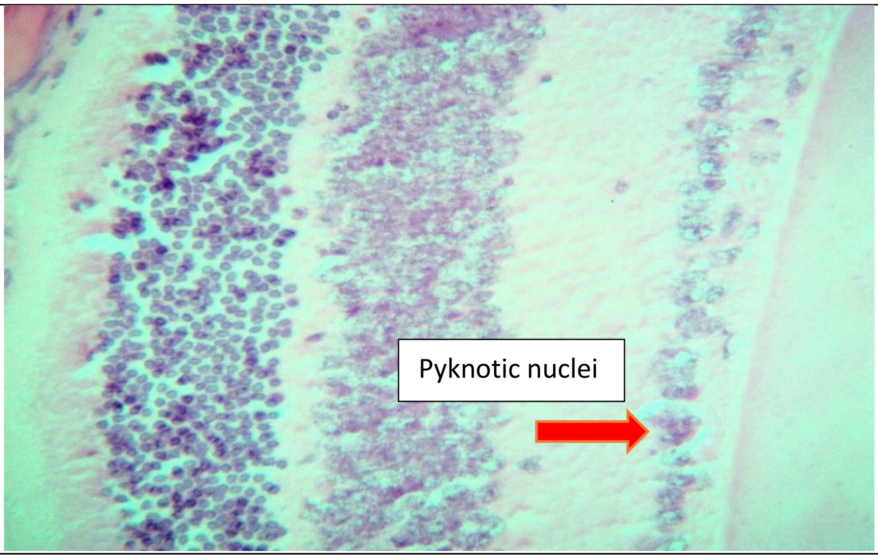
	Supplementary 1. Image shows disorganized ganglion cell layer and vacuolation (panel A) and pyknotic nuclei (panel B) at high power (400x).
